# Talking about Looks

**DOI:** 10.1007/s13164-017-0350-7

**Published:** 2017-07-04

**Authors:** Kathrin Glüer

**Affiliations:** Department of Philosophy, Stockholm University, Stockholm, Sweden

## Abstract

In natural language, looks-talk is used in a variety of ways. I investigate three uses of ‘looks’ that have traditionally been distinguished – epistemic, comparative, and phenomenal ‘looks’ – and endorse and develop considerations in support of the view that these amount to polysemy. Focusing on the phenomenal use of ‘looks’, I then investigate connections between its semantics, the content of visual experience, and the metaphysics of looks. I argue that phenomenal ‘looks’ is not a propositional attitude operator: We do not use it to ascribe propositional attitudes to subjects, but to directly ascribe looks to objects, where looks are relational properties. However, I go on to argue that, given the way we use phenomenal ‘looks’, these relational properties are ultimately best understood as phenomenal relational properties, i.e. in terms of relations involving experiences. Along the way, I endorse Byrne’s argument against Jackson’s claim that phenomenal ‘looks *F*’ only takes predicates for colour, shape, and distance, and raise the issue of compositionality for the resulting view according to which phenomenal ‘looks *F*’ is context-dependent in a way that allows it to take a vast range of predicates. I conclude by arguing that these considerations concerning the natural language use of ‘looks’, and in particular its phenomenal use, are water on the mills of phenomenal intentionalism, a position in the philosophy of perception according to which experiences are propositional attitudes with phenomenal looks-contents.

## Introduction

One morning in late summer, Alma looks out of the window of the cottage she and Martha have rented for their vacation. Alma looks at the old farm house across the meadow and the mountains beyond. “It looks as if the neighbours moved away a long time ago, doesn’t it”, she says, but Martha is in the kitchen making coffee and listening to the radio. “Let’s go hiking today”, she calls. The sun is shining but there are dark clouds gathering behind the mountains. “Absolutely,” Alma answers, “but it looks as if we will need our rain gear.” She walks into the kitchen. “What did you think of Bob?”, Martha asks. Bob is an old friend of hers they ran into at the country store the day before. “He looks like Mads Mikkelsen”, Alma says. “I liked the look of his sweater. Old-fashioned. A bit like the old abandoned farm over there.” She gestures in the direction of the house across the meadow. “It looked so red in that weird light.” Martha laughs, but then her face darkens. “It looks as if the stock market is crashing again”, she tells Alma while gesturing at the radio. They sit down at the kitchen table to listen. “We should switch to the other station,” Alma says, “they usually are more up to date.” They switch and learn that the situation has already much improved.

In this little story, there is a lot of talk about looks. Like everybody else, Alma and Martha are using looks-language in a variety of ways; they talk about the looks of objects and how they compare, but they also use ‘looks’ in their descriptions of the states of affairs that might or will obtain in the world around them. Such looks-language is the main topic of this paper. How do looks-locutions work, and what do they mean? Quite a bit has been said about this in the philosophical literature since the 1950s. Most, if not all, of this work has been in the context, and service, of the theory of perception. Claims about looks-language have been made to support a wide variety of accounts of (visual) perceptual experience, from sense data theory via the adverbial theory and intentionalism all the way to anti-intentionalist relational accounts.^1^

This paper will be no exception here – ultimately, I am interested in looks-language to see whether natural language does, or does not, furnish a ready-made sense of ‘looks’ that can be used to interpret one of the two central claims of a position I have been developing: “phenomenal intentionalism”. Phenomenal intentionalism combines the claim that perceptual experience is a subspecies of belief with the claim that visual experience takes what I have called *looks-* or *Lp-contents*, contents of the form *x looks F (to S at t)*, or *it looks as if p (to S at t)* (cf. Glüer 2009, 2014). It seems to me that previous investigations of looks-language, instructive and insightful as they are, have not always been looking at sufficiently varied examples from natural language. Despite all that has been said about the uses of ‘looks’, there still is a need, it seems to me, for careful exploration of, and theorizing about, looks-language. My aim in this paper is quite modest: To make some progress on these fronts.

I shall proceed as follows. In Section 2, I shall follow Chisholm and Jackson in distinguishing three main uses of ‘looks’: epistemic, comparative, and phenomenal uses. I shall explore these and their interrelations in some detail, and investigate whether their existence amounts to something like ambiguity or polysemy. In the sections to follow, I shall then focus exclusively on the third, or phenomenal, use of ‘looks’. In Section 3, I shall be concerned with its significance, if any, for the theory of perceptual experience. In particular, I shall argue that, prima facie at least, the existence of a third use is compatible with a wider range of positions on perception than one might expect. The only position directly threatened by the very existence of a third use appears to be Martin-style parsimony about looks. But I shall then go on to present a range of additional observations about the way phenomenal ‘looks’ is actually used in natural English. These, or so I shall argue, ultimately do speak both against construing it as a propositional attitude operator as well as against construing it as ascribing mind-independent, or non-phenomenal, relational properties to objects. These observations point towards the conclusion that the best semantics for phenomenal ‘looks’ treats looks as phenomenal relational properties. In the final section, I shall mainly investigate the range of predicates that can be modified by phenomenal ‘looks’, and what such combinations mean. I shall argue with Byrne and against Jackson that phenomenal ‘looks’ can take a vast range of predicates, and raise the issue of compositionality. I shall conclude that nothing in this paper prevents me from modelling looks-contents for experience on natural language phenomenal ‘looks’.

## The Uses of ‘Looks’

Following Chisholm (1957) and Jackson (1977), it has become customary in the literature to distinguish between at least three “uses” of ‘looks’ in natural English.^2^ The first of these is the *epistemic use*. Our initial story featured some quite typical examples of epistemic uses of looks:

1. It looks as if the neighbours moved away.
2. It looks as if we will need our rain gear today.
3. It looks as if the stock market is crashing.

When a speaker *S* uses ‘it looks as if *p*’ epistemically, *S* uses it to say something about their reasons or evidence for believing that *p*.^3^ According to what I shall call the “weak interpretation” of epistemic ‘looks’, speakers use it to say that they have (some) *reasons, or evidence,* for believing that *p*. According to the “strong interpretation”, just having reasons or evidence is not sufficient: what is required is *good, or undefeated,* reasons or evidence. Regardless of whether the weak or the strong interpretation is correct, there is a further distinction that is of interest here: that between *perceptual* and *non-perceptual* epistemic uses of ‘looks’. Epistemic ‘looks’ might, but doesn’t have to, indicate that the evidence for *p* is of a visual nature. This is the case for Alma’s utterances of (1) and (2). She looks out of the window and it is the way the old farm house across the meadow looks that is her reason for thinking it has been abandoned long ago. Similarly, the way the clouds behind the mountains look is her reason for thinking that it will rain later in the day. These are perceptual uses of epistemic ‘looks’.

The evidence indicated by epistemic uses of ‘looks’ need not be perceptual, however. Even though Martha’s evidence for thinking that the stock market is crashing comes through an auditory channel, its epistemic source is not perception, but testimony. Such non-perceptual epistemic uses of ‘looks’ most clearly show a feature of this use stressed by Brogaard (cf. Brogaard 2013, 4; 2015, 239): defeated epistemic looks “disappear”. Once Alma and Martha have switched channels and received a better evaluation of the situation on the stock market, the reasons they initially had for thinking that it was crashing have been defeated and the stock market no longer looks to be crashing. Let’s call this feature “defeated disappearance”.

If epistemic uses in general showed defeated disappearance, this would be strong evidence for the strong interpretation. Brogaard clearly thinks it is when she writes:

Epistemic ‘looks’ reports are evidence-bearing. It cannot epistemically look as if *p* if one’s total evidence indicates that not-*p*. (...) So, when epistemic ‘looks’ reports are accurate, they give us information about the speaker’s total subjective evidence (Brogaard 2013, 4).

But is it really the case that all epistemic looks disappear when defeated? It might look as if *perceptual* epistemic uses of ‘looks’ do not have this feature. Take Alma’s utterance of (1):

1. It looks as if the neighbours moved away.

And now imagine that Martha answers by saying “It certainly looks like that, but I happen to know that they have been hiding in the attic for years. They think the government is after them.” The example is essentially Jackson’s who observes that it is not inconsistent to say something like

(4) It looks as if the neighbours moved away, but I happen to know that they are hiding in the attic.^4^

The weak interpretation of epistemic ‘looks’ allows us to adopt Jackson’s description of such cases “as cases where we take it that though a certain body of visual evidence supports that *p*, other (non-visual) evidence makes it certain that not-*p*” (Jackson 1977, 31). So, in uttering (4), the idea is, we are saying that we have some visual reason or evidence for believing that the neighbours moved away, but that it is defeated by stronger evidence to the contrary. That there does not seem to be disappearance despite defeat here thus provides some support for the weak interpretation.

However, there *is* defeated disappearance even in “attic cases”. There is a strong contrast between (4) and (1) here. While (4) seems fine, asserting (1) after learning that the neighbours are hiding in the attic is just as unacceptable as asserting (3) after getting the better report about the stock market. This becomes very clear, I think, if you imagine that right after Martha tells Alma about the neighbours in the attic, Emma drops by and Alma tells her that it looks as if the neighbours moved away. It would be only natural for Martha to be somewhat upset about that. She might burst out with: “What? I just told you that they are hiding in the attic.” On the weak interpretation, that would not only be impolite, but quite unmotivated.

We might try to rescue the weak interpretation by construing defeated disappearance as some kind of implicature, for instance as a kind of scalar implicature. Just as saying that John has five children carries the implicature that he doesn’t have more than five, the idea is, saying that you have evidence for *p* carries the implicature that it is undefeated. But if implicature is the explanation for defeated disappearance, it should be possible to cancel that implicature. And while (4) could be taken as a case in point, the acceptability of sentences of the form

- (4 ^′^) It looks as if *p* but I happen to know that not-*p*.

appears to be restricted to perceptual uses. Analogous sentences featuring non-perceptual epistemic uses of ‘looks’ are much less acceptable, if at all. Imagine a variant of the stock market case in which Alma and Martha both hear the radio announcement of its crashing, and Martha comments:

(5) It looks as if the stock market is crashing, but I happen to know that it isn’t.

I think this is clearly unacceptable. All in all, it thus seems to me that the strong interpretation of epistemic ‘looks’ does somewhat better than the weak interpretation. But endorsing it leaves us with the question of how to make sense of the acceptability of sentences like (4).

A clue can be gotten, I think, from thinking about upset Martha again. She told Alma that the neighbours are hiding in the attic, but Alma nevertheless tells Emma that it looks as if they moved away. Alma can, it seems to me, defend herself by saying something like: “I didn’t say that they moved away, only that it *looks* that way”. But note that this works only if she *stresses* ‘looks’. And that suggests that what Alma exploits in her defense is in fact an ambiguity in ‘looks’ – it suggests that the use of ‘looks’ that makes (4) acceptable is *not* epistemic, after all. Rather, what we have here is a use of ‘looks’ describing the visual appearance of things. If that is correct, it would explain why sentences of the form (4 ^′^) are acceptable in perceptual cases, but not in cases involving clearly non-perceptual epistemic uses.

Which brings us to the two non-epistemic uses of ‘looks’ that are often being distinguished. Chisholm calls the second the “comparative use”. For that, too, we have some typical examples in our little story:

(6) Bob looks like Mads Mikkelsen.
(7) Bob’s sweater looks like the old farm house.

When we use ‘looks’ comparatively, the idea is, we compare things – in terms of their looks. More precisely, we say, or instance of Bob and Mads Mikkelsen, that the way they look is similar, or the same: They share a look. This need not be what we could call their “total look”, but can be a contextually salient aspect of that look. Thus, Bob’s sweater and the farm house presumably do not share their total look, but they do share a colour-look.

Such comparisons do not need to be expressed in terms of ‘looks like’. When Alma utters something like

(8) Bob’s sweater looks old-fashioned.

she is saying that the sweater has a look characteristic of old-fashioned things. And the things whose looks we compare don’t have to be material objects, or kinds thereof, either. Take (2) again:

(2) It looks as if we will need our rain gear today.

When Alma says that, she is most probably using ‘looks’ epistemically. But imagine her saying this while both she and Martha are looking out of the window. Then, she might just be drawing Martha’s attention to the fact that the scene outside has precisely that look that is so typical of impending rain.

Imagine now that Alma and Martha later learn that in the valley they are in now that look never indicates rain. The clouds always stay behind the mountains.^5^ Knowing this they cannot use ‘look’ epistemically in sincere assertions of (9):

(9) It looks as if it’s going to rain.

Using (9) comparatively also becomes more difficult – if at all, it will only work in contexts where it is clear that the comparison is with the look of impending rain at some other location, for instance, at home. But if Alma and Martha have a strong interest in talking about that dark-clouds-gathering-behind-the-mountains look, they might decide to give it a name – like I just did. Alma and Martha want something simpler, however, and decide to call that look Fjörgyn. From then on, when Alma wakes in the morning she’ll ask Martha: “Does it look fjörgyny outside?” Arguably, this is not a comparative use. It might not be English, either. We’ll get back to that.

For the simplest cases of comparative ‘looks’, cases where the comparison is between two individual objects like Bob and Mads Mikkelsen, we can make a first stab at providing the logical form of the relevant sentences:

- (6 ^′^) ∃*y* (WL*y* & has (Bob, *y*) & has (Mads, *y*)),

where ‘WL’ is a way of looking. Such sentences, that is, are quite naturally construed as quantifying over looks (or ways of looking). As it stands, (6 ^′^) is clearly inadequate, however, as not any old look would seem to be relevant. Rather, which look is at issue would seem to be a matter of contextual salience. Contextual salience will also be relevant for comparisons with the charactistic looks of kinds of objects, as in sentences like (8) or (10):

(10) That looks like a tomato.

(10) can plausibly be taken to say something like that there is a contextually salient way of looking that is characteristic of tomatoes and had by the demonstrated object. It thus can be construed as having a logical form like the following:

- (10 ^′^) ∃*y* (has(*o*, *y*) & (WL(*y*) & ID(C(*F*, WL, *c*), *y*))),

where ‘WL’ is a way of looking, *o* is the demonstrated object, ‘C(*F*, WL, *c*)’ the contextually salient way of looking characteristic of *F* s, and ‘ID’ is the identity function.

Construing comparative uses of ‘looks’ along lines such as these, we take them to be *quantifying over looks*. And we explain the comparative use of ‘looks’ in terms of looks, too; i.e. by *using* ‘looks’. This raises the questions of what the things quantified over in comparative looks-sentences are, and how to interpret ‘looks’ when used to explain what these sentences mean. *These uses*, it seems, cannot themselves be comparative uses (cf. Maund (1986) and Byrne (2009), 441). There seems to be a *third use*.

Chisholm and Jackson certainly thought there was a third use. Chisholm called it the “non-comparative use”, while Jackson called it the “phenomenal use”. These days, the terms “non-comparative” and “phenomenal” are often used interchangeably. But the range of expressions Chisholm thought could be used non-comparatively is significantly wider than those that according to Jackson are phenomenal uses. Even though I agree with Chisholm on the range of the third use of ‘looks’, I shall for the most part stick with the label “phenomenal use”, but initially without thereby buying into any particular interpretation of the third use. All I shall initially assume about this use is this: what we do when using ‘looks’ this way is *“directly” ascribing looks to things (or scenes)* – where “directly” is opposed to “by comparison”. What these looks *are* is precisely the question I want to initially leave open.

Typically, it is expressions of the form ‘looks *F*’ that we use phenomenally. In doing so, we typically say of something that it looks the *F*-way or that it has the *F*-look. Thus, in our initial story Alma said of Bob’s sweater that it looked red:

(11) Bob’s sweater looks red.

Other examples from Jackson (1977, 33) are:

(12) It looks triangular.
(13) The top line looks longer than the bottom line.

Chisholm, by contrast, also allows sentences like (14) to be used non-comparatively (cf. Chisholm (1957), 144):

(14) That looks centaurian.

But Jackson thought that the third use was “ tied to terms for colour, shape, and/or distance” (Jackson 1977, 33). Let’s call these the “basic sensible predicates”. While Jackson doesn’t explicitly spell out what being “tied to” these terms precisely means, he is usually taken to be claiming that combination with basic sensible terms is *both necessary and sufficient* for the phenomenal use of ‘looks’. According to Jackson, that is, it holds both that ‘looks’ can be used phenomenally only when combined with such terms and that whenever ‘looks’ is combined with such terms, it is used phenomenally.

This does not seem to be correct, however. If combination with basic sensible terms was necessary for a use of ‘looks’ to be phenomenal, ‘looks’ would be either comparative or epistemic in sentences like:

(15) The farmhouse looks old.

On the assumption that the different uses of ‘looks’ amount to something like ambiguity or polysemy, this would predict sentences like (16) to be unacceptable:

(16) The farmhouse looks red and old.

But they are not. In fact, most people find such sentences perfectly acceptable (cf. Thau 2002, 230, Byrne 2009, 442).^6^ This example shows that ‘looks’ can modify both ‘red’ and ‘old’ under one and the same interpretation. But which? If you think that ‘looks old’ cannot be phenomenal, this would mean that ‘looks red’ can be used either comparatively, or epistemically, or both. If you think, however, that ‘looks red’ can only be used phenomenally, you’d have to say that ‘looks old’ can be used phenomenally, too. You might of course also think that both ‘looks red’ and ‘looks old’ can be used in all three ways.^7^ In any case, the claim that the third use of ‘looks’ is “tied to” the basic sensible predicates appears to be false.

This might lead us to ask whether there is *any* form of looks-expression that can only be used a certain way. Maybe they *all* can be used any way we please? Brogaard (2013) argues that looks-expressions indeed are what we might call rather “versatile”. They can be put to all sorts of uses (in the right kind of scenarios). In particular, Brogaard argues that

(17) *x* looks *F*.

does not have to be phenomenal; it can be used both comparatively and epistemically. And that

(18) It looks like/as if *x* is *F*.

can be used phenomenally. Both claims seem correct to me.

Brogaard makes use of this versatility to argue that ‘looks’ is, in fact, polysemous. Her argument also assumes that defeated disappearance is a feature of epistemic uses of ‘looks’, and that it is a feature that phenomenal uses of ‘looks’ do *not* have. Imagine that you are looking at Tom, the tomato. Tom looks red to you. This provides you with a strong prima facie reason for believing that Tom is red. But now imagine that the following, so far hidden feature of the situation is revealed to you: Tom is in fact very cleverly lit by red light. Knowing this defeats your reason for believing that Tom is red. Given the colour of the light, Tom might just as well be white. You can even imagine that someone reliable tells or shows you that Tom in fact is white. This is what I shall call a “treacherous tomato scenario”. In such a scenario, the sentence

(19) Tom looks red,

remains true when construed phenomenally. (19), that is, remains true even after your reason for believing that Tom is red has been defeated. Phenomenal looks do not disappear when the evidence they provide has been defeated.

Brogaard’s argument for the polysemy of ‘looks’ then takes the form of a *reductio*. The argument employs a “coordination test”, i.e. proceeds by attempted conjunction reduction. Imagine an extended treacherous tomato scenario. Tom looks red but you know that he is white and his looking red is due to clever lighting. The extension is this: While looking at Tom you hear the radio reporting that the stock market is crashing. In this context, Brogaard submits, both of the following sentences are true:

(3) It looks as if the stock market is crashing.
(20) It looks as if Tom is red.

Because it is non-perceptual, (3) can only be read epistemically. (20) is most naturally read as epistemic, but then, it is false. But, Brogaard argues, a phenomenal reading of (20) is available. On this reading (20) is true. Now, on the assumption that the epistemic and the phenomenal use of ‘looks’ do *not* amount to polysemy, conjunction reduction should produce acceptable sentences even in scenarios like the extended treacherous tomato scenario. But

(21) *It looks as if (the stock market is crashing and Tom is red).

is *not* acceptable in this scenario. This provides some evidence for the claim that ‘looks’ is indeed polysemous.^8^^,^ 9

What about the phenomenal and the comparative use of ‘looks’, then – can we argue for the difference between them amounting to polysemy? Brogaard looks at examples like

(22) *The chair looks red and like a sofa.

which according to her are infelicitous. But this, Brogaard argues, is not due to any ambiguity in ‘looks’ itself.^10^ This is because the comparative use of looks is construed precisely as quantifying over phenomenal looks and therefore *analyzable* in terms of phenomenal ‘looks’.^11^ Now, I agree that the comparative use of ‘looks’ is to be analyzed in terms of phenomenal ‘looks’.^12^ What I don’t see is why that would mean that there is no polysemy here. After all, using ‘*x* looks *F*’ comparatively rather than phenomenally amounts to assigning a rather different logical form to the expression: Read that way, it not only contains (hidden) quantificational structure but also (hidden) context parameters. Each of these features by themselves would seem to carry the potential for inducing polysemy. Let’s consider the context dependence first. In the case at hand it pretty clearly induces a difference in truth conditions. Take

(23) *x* looks red.
(24) There is a contextually salient, characteristic way of looking that *x* shares with red things.

(23) and (24) are often made true by the very same look, but not always. Imagine that you are walking around in a very dimly lit environment. Everything around you looks some shade of grey or other. You learn to tell the red things apart from the others by the particular shade of grey they look in this environment. In such a scenario, there is a contextually salient look characteristic of the red things, but the red things do not phenomenally look red. Phenomenally, they look a particular shade of grey.^13^ This is a good reason to think that there is significant shift in the meaning of ‘looks’ between phenomenal and comparative uses, a shift that plausibly amounts to polysemy – even though the shifted meaning is analyzable in terms of the unshifted meaning.

Moreover, the difference in logical form between sentences construed as comparative and as phenomenal would just by itself seem to predict failing the coordination test. Assume that when read phenomenally, a sentence of the form *o looks F* is taken to directly ascribe the *F*-way of looking to *o*:

(25) Has(*o*, WLF),

where ‘WLF’ is that way of looking. Now take a sentence *o looks G* and read it comparatively. That results in something of the form

(26) ∃*y* (has(*o*, *y*) & (WL(*y*) & ID(C(*G*, WL, *c*), *y*))),

where ‘C(*G*, WL, *c*)’ is the contextually salient way of looking characteristic of *G* s. It should be clear that conjunction reduction on

(27) *o* looks *F* and *o* looks *G*,

where ‘looks *F*’ is phenomenal and ‘looks *G*’ is comparative won’t work. It is just not possible to get both *F* and *G* into the scope of ‘looks’ while holding on to reading one as phenomenal and the other as comparative. This, too, provides some reason to think that what we are dealing with here in fact is polysemy.^14^

If it is, we would expect to be able to actually produce infelicities when performing conjunction reduction on sentences of the form (27). But this is far from easy. In fact, we might easily get the impression that for pretty much any such sentence the result of conjunction reduction, i.e.

(28) *o* looks *F* and *G*

is just fine (cf. Breckenridge 2007, 41). Even if that were so, it just might be a consequence of the versatility of ‘looks *F*’ – as it can be used both phenomenally and comparatively for pretty much any *F*, there might simply always be *a* reading of sentences of the form (28) which is just fine. But in fact I think that things change once we consider admittedly rather special scenarios – scenarios where something might comparatively, but not phenomenally look *F*, for instance.

So, think of one of the scenarios we used above again: In a dimly lit environment, you tell the red things apart from those with other colours by means of the specific shade of grey they look. In such a scenario, it seems fine to say of something looking that shade of grey

(29) That one looks red.

Here, it is pretty clear that ‘looks red’ has to be taken comparatively. At the same time, you might of course say of the very same thing that it looks grey. But, it seems to me, you can’t felicitously say

(30) *That one looks red and grey.

Moreover, in scenarios like this we can also create the *lack of contradiction* that is another test for ambiguity. Thus it seems to me that I can without contradiction utter

(31) That one looks red but it doesn’t look *red*.

At least if there is a bit of stress on the second ‘red’ this seems fine to me. And it is clearly fine, I think, if we in addition to stress provide a bit of paraphrase for the comparative occurrence:

(31+) That one looks red – just like the other red things around here – but it doesn’t look *red*.

There is thus at least some reason to think that the three uses of looks tradition distinguishes between not only actually exist, but also that this amounts to something like polysemy in ‘looks’. I don’t take these considerations to be conclusive, but I do hope that they can help a bit to move the debate surrounding the uses of ‘looks’ forward. In what follows, I shall be solely concerned with the phenomenal use of ‘looks’. In the next section, I shall have a look at possible connections between the phenomenal use of ‘looks’ in natural language, the content of visual experience, and the metaphysics of looks.

## ‘Looks’, Looks, and Visual Experiences

A natural idea about the phenomenal sense of ‘looks’ is the following: Phenomenal ‘looks’ can be used for reporting visual experience. In telling us how things phenomenally look, the idea is, speakers (typically) report on how they visually experience them or what the world around them is like – according to their visual experience. This chimes well with the fact that we typically talk about looks in situations where these are misleading, or where we suspect them to be misleading. Thus, John in Sellars’s classical story first learns to use ‘looks *F*’-locutions in a scenario where he has to pick a tie by colour but under dubious lighting conditions (cf. Sellars 1963, 142ff). According to Sellars, what John learns when he learns to use the sentence ‘this necktie looks green to me’ is “a way of reporting an experience” (Sellars 1963, 146), more precisely, a way of reporting an experience that “as an experience” is identical to a seeing that something is green (ibid., 145). It thus seems natural to think that phenomenal looks-reports not only report visual experience, but also that they are sensitive to precisely the *phenomenal* characteristics of these experiences.

In present day philosophy of perception, one of the big divides is between what I shall call “intentionalism” and “anti-intentionalism”. Intentionalists and anti-intentionalists agree that perceptual experiences are (conscious) mental states with distinctive (sensory) phenomenal character. They tend to disagree on the nature of phenomenal character, but the crucial difference concerns the question of *content*.

*Intentionalism* is any position that construes perceptual experience as a mental state with representational content. As I shall use the terms here – in a wide and very uncontentious sense – this amounts to construing perceptual experience as a propositional attitude.^15^ According to standard intentionalism, experiences ascribe sensible properties to ordinary material objects. An experience as of something *F* thus has a content of the form *x is F*. There is a lot of discussion about further details, in particular about whether these contents are singular or general, but I shall for the most part abstract from that here. As I said right at the beginning, my own form of intentionalism – phenomenal intentionalism – does not construe experience as having such standard contents. According to phenomenal intentionalism, visual experience in particular has “looks-contents”, i.e. contents of the form *x looks F*. Intentionalism is often combined with an explanatory ambition regarding phenomenal character: What is usually called “representationalism” is the claim that the representational content of perceptual experience determines, and thus explains, its phenomenal character.^16^

*Anti-intentionalism* on the other hand denies that experience has representational content. It mostly comes in the form of *relationalism*. According to relationalism, veridical perceptual experiences are relations between subjects and (mind-independent) objects. Here, the object of a veridical experience is a constituent of the experience. Anti-intentionalists have a tendency to deny the existence at least of any interesting phenomenal use of ‘looks’, while intentionalists mostly are happy to simply assume its existence.^17^ This is slightly curious – as it is at least not immediately obvious what exactly motivates these tendencies.

One way of making aconnection goes via aparticular challenge to intentionalism – the “indeterminacy challenge”. Recent discussion of it has been centered on Travis (2004), but you can find all the elements to the challenge in Austin (1962). Travis claims that experience content is not “looks-indexed”: there is no way to read off the presumed contents of experience from the way we ascribe looks to, or perceive, things. Looks, that is, do not determine experience contents (cf. Travis 2004, 63ff). This is supposed to be achallenge, Itake it, because of abackground assumption to the effect that if experiences did have contents, they would (have to) be so determined. Ishall not discuss the plausibility of this assumption. What interests me right now is acertain response intentionalists have given to the indeterminacy challenge. Byrne, for instance, explicitly connects the indeterminacy challenge to Jackson’s discussion of phenomenal ‘looks’ and his argument that its analysis leads to sense data (cf. Jackson 33). Byrne comments:

With hindsight, [Jackson] could have taken it to index the content of perception instead: if *o* looks _*p*_ (the subscript indicating the phenomenal use) *F* to *S* then *S* exes, of *o*, that it is *F* (Byrne 2009, 442).

‘Exing’ is Byrne’s term of art for the experiential propositional attitude.^18^ The “indexing principle” Byrne suggests (on Jacksons behalf) thus is following:

(I) An experience report of the form *o looks*_*p*_ *F* (to *S*) is true iff *S* exes that *o* is *F*.

Byrne himself does not subscribe to this principle (see fn. 34 for why). But others seem quite ready to embrace it. Schellenberg certainly seems to endorse what Ishall call “standard indexing” when she writes:

The force of the indeterminacy objection relies on ‘looks’ being understood comparatively. If ‘looks’ is understood noncomparatively, (...) then the way things look fixes the content of experience (Schellenberg 2011, 9).

And so does Brogaard:

Representationalism predicts that how things phenomenally look to the subject reflects the content of the experience (Brogaard 2010, 373).^19^

Intentionalists employing something like the standard indexing principle in response to the indeterminacy challenge construe the phenomenal properties that looks-reports are sensitive to as properties the experience has in virtue of its content. So construed, a looks-report does not commit the reporter to the truth of the reported experience’s content – to *o*’s being *F* – but only to *S*’s having an experience with the content that *o* is *F*. Employing the standard indexing principle thus, in effect, amounts to treating phenomenal ‘looks’ as a propositional attitude operator: ‘*o* looks *F* (to *S*)’ is treated as equivalent to ‘It looks (to *S*) as if *o* is *F*’ and both are construed as true iff *S* exes that *o* is *F*, where ‘exes that’ is a propositional attitude operator just like ‘believes that’. Let’s call proposals along these lines “attitude operator semantics” for phenomenal ‘looks’.

If phenomenal ‘looks’ actually works this way in natural language that would be very good evidence at least for the claim that pre-theoretically, we conceive of perceptual experience as (what a philosopher would call) a propositional attitude. In other words: If phenomenal ‘looks’ in natural language indeed is a propositional attitude operator, that is (at least some) evidence for intentionalism. Of course, we can have good reasons to give up on (parts of) our pre-theoretical conceptions of pretty much anything – but if something is so deeply engrained as to be encoded in the syntax and semantics of natural language, giving it up is a rather high cost. The anti-intentionalist would be able to avoid paying this price if they instead could convince us that there is no phenomenal use of ‘looks’.

But while attitude operator semantics certainly is not lacking prima facie plausibility, it is by no means clear that it is the best way to go. Other options become more visible once we think in terms of the metaphysics of looks. According to attitude operator semantics, looks presumably are experiences. Or maybe they could be construed as properties things only have as experienced: An object has a look iff it is the object of someone’s experience. This isn’t implausible, but neither is it implausible to think that things have looks even if no-one is looking (someone who makes heavy weather of this is Martin (2010)). Of course, some feel that such properties are best understood in terms of dispositions of objects to look certain ways if someone were to look at them. But others feel that looks are more “objective” than that, i.e. that they are mind-independent properties of objects. Let’s call all accounts of looks according to which looks are mind-independent properties of objects “non-phenomenal accounts”.

According to Noë, for instance, looks are what he calls “perspectival properties”, or “P-properties” (cf. Noë 2004, 84). These are relational properties of objects, but they are not relations to experiences. “P-properties are, in effect, relations between objects and their environment”, Noë explains (Noë 2004, 83). P-shapes, for instance, are determined by an object’s shape and its relation to the location of a (possible) perceiver. More precisely, P-shapes are occlusion shapes: “[t]he P-shape is the shape of the patch needed to occlude the object on a plane perpendicular to the line of sight” (Noë 2004, 83). If P-properties are looks, it is only natural to expect there to be a use of ‘looks’ that, in effect, ascribes P-properties to objects. Thus, attitude operator semantics would seem to have a rival: “P-property semantics”.^20^ We could thus have a “relational semantics” for the third, or phenomenal, use of ‘looks’ without adopting a phenomenal account of looks – where a phenomenal account of looks construes looks as relational properties involving experiences.

Favoring relational semantics over attitude operator semantics for ‘looks’ does not prevent us from hanging on to the idea that phenomenal ‘looks’ reports experiences (and their content). Noë, for one, thinks that P-properties are represented in the content of experience (along with intrinsic properties of objects). And according to phenomenal intentionalism, all experience contents are looks-contents, so phenomenal ‘looks’ can indeed be used to report experience.^21^

But why would we want to construe looks as relational properties in the first place? One observation naturally taken to support relational accounts is the following: They promise an explanation of the fact that intuitively, the truth value of utterances of sentences like (32) and (33) varies with the circumstances with respect to which they are evaluated:

(32) Sam looks straight.
(33) Sam looks bent.

Sam is a straight stick. Looking at Sam in broad daylight with nothing obscuring our view, an utterance of (32) strikes us as true, while (33) seems false. But looking at Sam half-way immersed in a glass of water, (32) strikes us as false, while an utterance of (33) seems true. It is natural to think that these truth values reflect changes in looks: Sam’s look changes when Sam is put into water. Since there is no change in Sam’s intrinsic properties, it is tempting to further analyse the change in look as a change in Sam’s relational properties. Such context-dependence does provide support for thinking of looks as relational, but it does not, by itself, favour either a phenomenal or a non-phenomenal account of them.

As far as I can see, it is only the adoption of the most extreme non-phenomenal parsimony regarding looks that is hard to reconcile with the existence of a third use of ‘looks’, a use of ‘looks’ interpreted as directly ascribing looks to objects. Martin suggests identifying looks with intrinsic properties of objects, more precisely, with their basic visible properties (such as shape and color) (cf. Martin 2010, 161; 207ff). Directly ascribing a looks-property such as looking bent to Sam then simply amounts to ascribing bentness to him. A “parsimonious semantics” for phenomenal ‘looks’, that is, cannot explain the intuitive truth-value changes for sentences like (32) and (33). In order to have a chance at making a parsimonous account of looks compatible with the intuitive context-dependence of the truth values of looks-sentences, we need to make it plausible that virtually none of them are direct ascriptions of looks to objects. Rather, they need to be construed as comparative.^22^

With the exception of extreme parsimony about looks, then, there are, it seems, various accounts of looks available that could inspire prima facie quite plausible interpretations of phenomenal ‘looks’, and among them are interpretations that do not construe ‘looks’ as an attitude operator. Is it, then, only the most extreme relationalist that really has anything to fear from the existence of a third, or phenomenal, use of ‘looks’? I ultimately don’t think so. But to make any further progress on how best to interpret phenomenal ‘looks’, we need to try a new tack. For all that has been written about the uses of ‘looks’, we still know too little about how ‘looks’ is actually used in natural language. In what follows, I shall draw attention to certain patterns in the usage of phenomenal ‘looks’ that are hard to reconcile not only with attitude operator semantics but also with non-phenomenal semantics. Again, the observations I shall present will hopefully push the debate forward, but hardly clinch it.

One relevant set of observations concerns that-clauses. Typically, propositional attitude operators take that-clauses. ‘Looks’ does not. Take Tom, the tomato. We can use (34) to say something about Tom’s look. And we can report *seeing that* Tom is red. When we say that we see that Tom is red, we presumably report that we believe or know Tom be red where that belief or knowledge is based on visual experience of Tom. But trying to report on that experience by means of ‘looks that’ isn’t even grammatical:

(34) Tom looks red (to *S*).
(35) *It looks (to *S*) that Tom is red.
(36) *S* sees that Tom is red.

This holds across all the modality-specific appear verbs:

(37) Tom smells/feels/tastes/sounds ripe (to *S*).
(38) *It smells/feels/tastes/sounds (to *S*) that Tom is ripe.
(39) *S smells/feels/tastes that Tom is ripe.

It does not hold for ‘seem’ and ‘appear’, however:

(40) Tom seems/appears ripe (to *S*).
(41) It seems/appears (to *S*) that Tom is ripe.

Taking that-clauses is sufficiently typical for propositional attitude operators, it seems to me, for this observation to be something that the proponent of attitude operator semantics for phenomenal ‘looks’ would want to explain, or explain away. It might only be a minor perplexity, but still.^23^

The following set of observations, however, I take to be quite serious for attitude operator semantics. We start by observing something that initially seems to speak for this interpretation: We do get *substitution failure* in looks-contexts. Imagine that you look at the sky outside your window. Fairly close by, a figure in Superman-outfit is zooming by. Intuitively, you can now truly say

(42) It looks as if Superman is flying by.

Nevertheless, it might strike you as clearly false to describe the situation by means of (43):

(43) It looks as if Clark Kent is flying by.

So, we can get truth-value change as a result of substituting co-referential expressions in looks-contexts. Which looks like the kind of *intensionality* you would expect of a propositional attitude context.^24^

But the hypothesis that ‘looks’ is a propositional attitude operator is not the only possible explanation for such substitution failure. And in fact, I think that there is a better explanation for the substitution behaviour of looks-contexts: My hypothesis is that in looks-contexts what matters for substitutability is not co-intensionality (or even co-hyperintensionality) but a property I have called “co-phenomenality” (cf. Glüer 2014, 2016a). More precisely, the hypothesis is:

(CP) In phenomenal looks contexts, co-phenomenal expressions can be substituted salva veritate.

Co-phenomenality is a pretty weird property: It is different from, and independent of, both co-extensionality and co-intensionality. Expressions can be co-phenomenal without even being co-extensional. In that case, ‘looks’ would behave in a way very different from any known propositional attitude operator. In fact, it would behave in a way no propositional attitude operator *should* behave.^25^ And it does.

To illustrate what co-phenomenality is, let’s look at a range of sentence (schema) pairs.

(44) *o* looks green.
(45) *o* looks triangular.

Clearly, ‘green’ and ‘triangular’ are not co-phenomenal. The look ascribed to *o* by means of (44) and (45) is very different. That holds even in a world where everything green is triangular, and vice versa. Even in such a world, these looks can come apart. That might not be the case for necessarily co-extensional predicates, however. Take these:

(45) *o* looks triangular.
(46) *o* looks trilateral.

It might be the case that it holds for all “phenomenal” predicates – i.e. predicates the modification of which by phenomenal ‘looks’ results in a meaningful expression – that necessary co-extensionality does amount to co-phenomenality.

As long as we stick to the basic sensible predicates, we don’t get much co-phenomenality. We do get relations of entailment. These appear to correspond to determinable-determinate relations. Thus, (48) entails (47), but not the other way. (47) ascribes a more determinable look.

(47) *o* looks red.
(48) *o* looks red _52_.

We do get much more co-phenomenality once we leave the basic sensible predicates. But I shall get back to that in the next section. For the purposes of this section, two more observations are crucial.

The first concerns expressions other than predicates. Co-phenomenality is not confined to predicates; proper names, for instance, can be co-phenomenal, too. Take Tom, the tomato, again. Tom has an identical twin, Tim. There is no way Tom and Tim can be told apart by their looks alone. Even side by side in bright daylight you can’t tell which is which – unless they wear their name tags. Now imagine looking at one of them sitting on the kitchen table right in front of you. You can equally well (and truly) describe the way things look to you by either (49) or (50):^26^

(49) It looks as if Tom is on the table in front of me.
(50) It looks as if Tim is on the table in front of me.

For those who know Tom and Tim, ‘Tom’ and ‘Tim’ are co-phenomenal. The Tom-look is the same as the Tim-look. Thus, even expressions that are not even co-referential can be co-phenomenal. Not all of them are, of course. That’s where we came in: ‘Superman’ and ‘Clark Kent’ aren’t co-phenomenal; the Superman-look is very different from the Clark Kent-look.

The observations made so far already provide a good case against attitude operator semantics. Phenomenal ‘looks’ allows for salva veritate substitutions that no propositional attitude operator should allow. At this point, it therefore looks as if the phenomenal use of ‘looks’ is better interpreted as ascribing relational properties to things rather than as ascribing propositional attitudes to subjects. But which? Should we go for a phenomenal or for a non-phenomenal semantics for the third use of ‘looks’?

As we saw above, both phenomenal and non-phenomenal semantics deals equally well with the context-dependent truth value changes we observe in sentences like

(33) Sam looks bent.

Sam’s looking bent can, for instance, be interpreted along Noë’s lines as Sam’s having a bent occlusion shape (as he does when half-way immersed in water).

But occlusion shapes do not explain all relevant truth value changes. Take Dubbit, the duck-rabbit. And consider the following two sentences:^27^

(51) Dubbit looks rabbity.
(52) Dubbit looks ducky.

By nature, Dubbit is such that she can look both rabbity and ducky. Some people want to say that, indeed, Dubbit has both looks simultaneously. But even for those feeling that temptation, there is no single time *t* and subject *S* such that both

(51+) Dubbit looks rabbity to *S* at *t*.
(52+) Dubbit looks ducky to *S* at *t*.

are true. The looks making true (51+) or (52+) are what I have elsewhere called “fully gestalted looks”, i.e. ways things look after possible Gestalt switches have taken place (cf. Glüer 2016a). Sentences like these are subject to truth value changes just like sentences like (33), but these changes are not functions of the same context parameters. Dubbit’s Gestalt switches are, for instance, not a function of changes in Dubbit’s occlusion shape. These changes in an object’s look, it seems to me, cannot be explained in terms of the object’s relations to its environment at all. None of Dubbit’s non-phenomenal relational properties need to change for Dubbit to undergo a Gestalt switch.^28^

What’s more, an object doesn’t need to be an “ambiguous figure” – something like a duck-rabbit or a Necker-cube – in order to undergo changes in looks that have nothing to do with changes in its P-properties (or any other non-phenomenal relational properties). Painters learn to make pretty much any old object undergo a certain kind of overall Gestalt switch: They learn to make objects have what is sometimes called “painter’s looks”. An object’s painter’s look is a “flattened Gestalt”: Having it’s painter’s look, an object looks like a paper cut-out, i.e. as if it was a very thin, flat object perpendicular to your line of sight. Learning to see objects this way involves learning to switch Gestalt also on all the depth clues they typically provide. Thus, what typically looks like a shadow will look like an difference in objective colour when an object has its painter’s look. These changes cannot be understood as changes in an object’s non-phenomenal relational properties. No differences in looks that result from nothing but mere Gestalt switches can, and pretty much any object can (be made to) switch Gestalt.^29^ Phenomenal ‘looks’ clearly is sensitive to changes in look that result from mere Gestalt switches, not just to changes that result from differences in an object’s relations to its environment. Ultimately, a phenomenal semantics for phenomenal ‘looks’ therefore has a bit of an edge over a non-phenomenal semantics.

To sum up: In this section I have argued that phenomenal ‘looks’ is not a propositional attitude operator. When we use phenomenal ‘looks’ in natural language, we do not ascribe experiential attitudes to subjects; rather, what we seem to be doing is ascribe relational properties to the objects of experiential attitudes.^30^ These properties in turn are best construed as phenomenal relational properties, i.e. in terms of relations between objects and the experiences they are the objects of.^31^ Therefore, it seems to me, the third use of ‘looks’ is aptly named the *phenomenal* use, after all. In the next and final section, I shall explore its (syntax and) semantics just a bit further.

## More on Phenomenal ‘Looks’

If phenomenal ‘looks’ is not a propositional attitude operator, what is it, then? Is it a sentential operator at all? Or rather a predicate modifier? On the assumption that sentences of both of the following forms can be used phenomenally, it would prima facie seem to be able to be both:

(23) *x* looks red.
(18) It looks like/as if *x* is *F*.

This raises the question of the relation between ‘looks’ as occurring in these constructions. Brogaard (2013, 2015) argues that ‘looks’ is a subject-raising verb. She looks at sentences of the following forms:

(53) *o* looks *F*.
(54) *o* looks to be *F*.
(55) It looks as if *o* is *F*.

According to her, (53) results from (54) by infinitive deletion. And (54) results from (55) by means of subject raising. According to Brogaard, then, ‘looks’ is a sentential operator, but one that can, on the surface, look like a predicate modifier. If this is right, then ‘Tom looks (to be) red’ and ‘It looks as if Tom is red’, when read phenomenally, are synonymous.^32^

What Brogaard does not tell us, however, is how to think of phenomenal looks-sentences containing determiner phrases instead of singular terms – such as ‘something looks *F*’ or ‘it looks as if some *G* is *F*’ – or sentences containing quantifier phrases such as ‘it looks as if there is something that is *F*’ or ‘There is something that looks *F*’. In both kinds of cases, the sentence that results from “exporting” the determiner phrase/quantifier out of the scope of ‘looks’ is intuitively *not* synonymous with the original:

(56) It looks as if something is *F*.
(57) Something looks to be *F*.

By uttering a sentence of form (57) you incur an existential commitment that uttering one of form (56) does not carry. It is therefore at least not obvious how Brogaard’s suggestion is to be generalized to all the relevant looks-sentences.

For the semantics of visual experience, I have suggested treating the sentential operator *L*_1_(*p*) as analytically primary, and to define a predicate modifier *L*_2_(*F*) in terms of it. Applying this suggestion to natural language phenomenal ‘looks’, (56) would be further analyzed as being of the form

- (56 ^′^) *L*_1_ (∃*x* (*F* *x* ))

And then, the predicate modifier *L*_2_ can be defined as follows:

(L) ∃*x* ((*L*_2_(*F*))(*x*)) ≡_*df*_∃*x* *L*_1_(*F* *x*)

That is, we would define *L*_2_ in terms of *L*_1_ in precisely the case where the quantifier has already been exported out of the scope of *L*_1_ – and the existential commitment has already been incurred. This leaves (56 ^′^) itself free from such commitment (cf. Glüer 2009). Whether this suggestion ultimately works for natural language phenomenal ‘looks’ is, of course, a topic to be further investigated. We haven’t come across anything in the course of the present investigation that would make its applicability unlikely, and that is where I shall leave the issue for now.

I would like to conclude by raising a further issue regarding the semantics of phenomenal ‘looks *F*’. Let’s start by revisiting Jackson’s claim that phenomenal ‘looks *F*’ takes only basic sensible predicates. As already mentioned, Byrne (2009) takes issue with this claim. He argues that a sentence like

(16) The farmhouse looks red and old.

can not only be read epistemically and comparatively, but also phenomenally.^33^ And that of course means that sentences of the form

(15) The farmhouse looks old.

can be read phenomenally. But how, precisely? Consider first a comparative reading: We are saying of the farmhouse that it has a contextually salient look, a look characteristic of a certain kind of old thing – old houses, say. Identifying that look might require some background knowledge on the part of the hearer. But anyone familiar with that look, Byrne’s idea is, can by means of (15) also directly ascribe precisely that look. This seems right to me. Just as Alma and Martha named the clouds-behind-the-mountain look ‘Fjörgyn’ and subsequently used ‘looks fjörgyny’ to directly ascribe that look to scenes in front of their eyes, Byrne suggests, we in fact do use all sorts of ‘looks *F*’ constructions this very way.

An even more intriguing example Byrne uses is that of a naked mole rat. Naked mole rats are fascinating underground creatures living in colonies on the outskirts of the Sahara. Their societies are organized like those of ants or bees, ruled by an oversized queen, and they build vast systems of narrow tunnels in which they run around around at great speed – both backwards and forwards. What interests us here is the way they look. Their skin is almost hairless, pinkish, and very wrinkled. They look – old. Mora is one of them.

(58) Mora looks old.

When using (58) comparatively, Byrne suggests, we say that Mora has a contextually salient look characteristic of (certain) old things. And again, we can use (58) phenomenally and ascribe this look directly to Mora as well.^34^ Byrne seems right to me on this, too. What complicates matters here is, of course, that the old-look we ascribe to Mora is different from the old-look we ascribed to the farmhouse. Which it is seems to be a matter of the context: In a context where we are looking at Mora, the salient old look is one typical of pink-skinned old creatures, while it is one typical of houses in the farmhouse context. But that does not prevent us from ascribing either of them directly to Mora or the farmhouse. In each case, what we ascribe is the salient old-look.^35^

Moreover, we seem to be able to ascribe the very same old-look to Mora by means of basic sensible predicates – as in

(59) Mora looks bald, pink, and wrinkled.

In general, it seems like there is very little, or even no, context dependence for the looks picked out by means of ‘looks *F*’ where *F* is a basic sensible predicate. We might even hypothesise that for every meaningful ‘looks G’ where *G* is not a basic sensible predicate, we should at least in principle be able to form a (possibly very) complex basic sensible predicate (such as ‘bald, pink, and wrinkled’) that can be used to ascribe the same look.

Be that as it may, what interests me now is how precisely the combination of ‘looks’ with apredicate is supposed to work. Byrne does not consider this in great detail, but after considering some further expressions – ‘looks Scandinavian’ and ‘looks centaurian’ – he suggests thinking of such combinations on the model of idiomatic expressions such as ‘red hair’:

Similarly, if someone looks _*p*_ Scandinavian, and so looks to have the stereotypical Scandinavian bodily features (straight blond hair, small nose, pale skin, etc.), he can be as he looks _*p*_ without being Scandinavian. (...) [T]hat animal, which looks _*p*_ centaurian, can be as it looks _*p*_ without being acentaur. ‘Looks _*p*_ *F*’ is therefore idiomatic in the interesting way ‘red hair’ is. ‘Red hair’ does refer to hair of adistinctive colour similar to red (and so is an example of polysemy), but that orangeish shade is not the semantic value of ‘red’. (‘Looks _*p*_ Scandinavian’ and ‘red hair’ are thus quite different from paradigmatic idioms like ‘blue blood’ and ‘green thumb’) (Byrne (2009), 444, subscripts changed).

If phenomenal ‘looks *F*’ indeed ascribes the salient *F*-look to an object, then, it seems to me, Byrne has to be right in thinking that ‘red’ in ‘looks red’ does not have the semantic value it normally has. It is, after all, not the look that is red.

However, I also think that it would be quite unfortunate if we had to settle for the conclusion that phenomenal ‘looks *F*’ is idiomatic – even in the interesting way ‘red hair’ is. Already on the basis of the examples we have looked at, it seems safe to predict that phenomenal ‘looks’ can modify a very large range of predicates. Moreover, the semantic effects of such combination seem to be quite systematic. All of this will be hard to explain if we go idiomatic on phenomenal ‘looks *F*’. We should also not forget the peculiar substitution behaviour we observed earlier. This presumably would receive no explanation at all on an idiom account.

What a large range of meaningful combinations with systematic effects would seem to call for is, of course, a compositional semantics predicting those effects. But a (classically) compositional semantics is precisely what we cannot have if ‘*F*’ does not have its usual semantic value when combined with phenomenal ‘looks’. This is not as much of a dilemma as it might look to be, however. For even if we cannot have a semantics that is classically compositional, we might be able to have one that is *general compositional* (cf. Pagin and Westerståhl (2010a, 2010b)). And that is just as good: It gives precisely the kind of explanation of the semantic effects of composition that we would like to see.

My hypothesis regarding phenomenal ‘looks *F*’ is the following

(S) If two predicates ‘*F*’ and ‘*G*’ are co-phenomenal (in a context *c*), then phenomenal ‘looks *F*’ and ‘looks *G*’ are synonymous (in *c*).

If that is correct, and generalises to other forms phenomenal looks-sentences can take, it explains the peculiar substitution behaviour in phenomenal looks contexts that we observed earlier.

What we need next is a systematic semantic explanation of the synonymy. The basic idea would be the following: phenomenal ‘looks’ is a “semantic evaluation switcher”.^36^ A phenomenal looks-modified predicate *F* does not denote *F*-ness but rather phenomenal *F*-ness. When in the scope of phenomenal ‘looks’, that is, a predicate *F* is evaluated by means of a function assigning sets of phenomenal *F* s (at worlds and contexts) as its extensions – instead of sets of *F* s. Such a switcher semantics is not classically compositional, but it is general compositional. Obviously, this is the merest sketch of the idea, which I hope to develop in much more detail in a paper of its own.

This paper, I would like to round off by revisiting the propositional attitude operator question. For even if we find the substitution behaviour of phenomenal ‘looks’ peculiar, we might wonder whether phenomenal looks contexts are really so very different from, for instance, belief contexts. After all, there is much a speaker needs to know besides their meanings to understand that two expressions can be substituted salva veritate in a phenomenal looks context. This is especially important if phenomenal ‘looks’ indeed meaningfully combines with lots of predicates over and above the basic sensible ones. One might therefore wonder whether there really is any deep difference here. After all, belief contexts allow for all sorts of peculiar substitutions, too – provided the speaker has sufficient extra-linguistic knowledge. And isn’t that just knowledge of the very kind required for substituting into phenomenal contexts, too?^37^

While it is definitely true that a sufficient amount of “worldly” knowledge will make it possible to substitute expressions in belief contexts that do not have the same (hyper-)intension, I nevertheless think that a closer look at the kind of knowledge required reveals that what is going on with phenomenal contexts really is fundamentally different. Take a belief sentence of the form

(60) Bob believes that *o* is *F*.

Assume that *F* and *G* are co-extensional only in special contexts of kind *k*. What would we need to know to substitute *G* for *F* in (60)? More precisely, what would we need to know in order to know that we can do so without changing (60)’s truth value? We would need to know that *F* and *G* are co-extensional in *k* contexts, and that the context is of kind *k*. But we would *also* need to know a lot about Bob’s further beliefs, other propositional attitudes, and degree of rationality. This is just what is to be expected of a propositional attitude context. It is not at all peculiar – but it is really messy. Substitutivity in phenomenal contexts, by contrast, might be peculiar, but it is not messy. As noted above, it is really not messy at all, but rather as neat and systematic as the reference of personal pronouns. Which *F*-look is attributed by means of ‘looks *F*’ is straightforwardly determined by the context of utterance: It’s the salient *F*-look. Phenomenal ‘looks’ really doesn’t behave the way a well brought up propositional attitude operator behaves.

What does all of this mean for the theory of perceptual experience? To the extent that phenomenal ‘looks’s being a propositional attitude operator in natural language would provide support for standard intentionalism, this is bad news for standard intentionalism. Its not being a propositional attitude operator, however, does not favor anti-intentionalism over intentionalism in general: If anything, it is water on the mills of phenomenal intentionalism.

## Footnotes

1 Just a few examples for illustration: Jackson (1977) uses claims about looks-language to support a sense datum theory, Chisholm (1957) to argue for adverbialism, Alston (2002) for the theory of appearing, Sellars (1963), Byrne (2009), and Brogaard (2013, 2015) for intentionalism, and Martin (2010) claims that there are no semantic arguments against identifying looks with basic sensible properties such as redness and roundness, thereby providing support for a form of anti-intentionalist relationalism.

2 I cannot go through all that has been written on this topic here. For useful surveys, cf. Brogaard (2014, 2015), and Breckenridge (2007), ch. 2.

3 Epistemic ‘looks’ thus looks quite a bit like a kind of epistemic modal. I tend to think that it, in fact, is one. For a slightly different take, cf. Brogaard (2015).

4 Cf. Jackson (1977), 31. Jackson here is discussing the idea that epistemic ‘looks’ should be understood in terms of “tentative, guarded, etc. assent”, but as (rational) assent is based on total evidence, his observations are equally relevant to the question of whether epistemic ‘looks’ is best interpreted weakly or strongly.

5 The example again is one of Jackson’s, cf. Jackson (1977), 31.

6 I have used this example numerous times, and nobody has ever found it anomalous – except for Peter Pagin.

7 Thus, Byrne argues that both ‘looks red’ and ‘looks old’ can be used in all three ways. It seems to me that he is right about this. We’ll get back to this in Section 4.

8 Some, but very few, people report not being able to read (20) any way other than epistemically. If you are one of those, try

(i) *The stock market looks to be crashing and Tom to be red.

instead. I think that ‘the stock market looks to be crashing’ can be read epistemically, and indeed has to be in the extended treacherous tomato scenario. I also think that in this scenario (i) is not acceptable.

9 On the assumption that comparative uses quantify over phenomenal looks, we would predict that comparative looks do not disappear when defeated, either. As far as I can tell, that is the case. On the further assumption that (20) can be read comparatively, we can run an analogous argument for the claim that the distinction between epistemic and comparative uses of ‘looks’ amounts to polysemy.

10 Rather, the ambiguity here is – somehow – created by the combination with ‘like’ (cf. Brogaard 2013, 20f).

11 For other, structurally somewhat similar arguments against ambiguity in ‘looks’, see Thau (2002), 229ff; Breckenridge (2007).

12 The same holds for *perceptual* epistemic uses of ‘looks’.

13 In Section 4, I will argue that even phenomenal ‘looks *F*’ comes with a (hidden) context parameter and means something like the salient red-look. But, or so I shall also argue, at least for basic sensible predicates such as ‘red’, the resulting range of looks they can be made to denote when looks-modified will be very limited. It certainly will not include shades of grey.

14 Breckenridge (2007) suggests an event-semantics for ‘looks’ according to which looks are events and ‘looks *F*’ is a definite description of a property of such events: the way *F* s look. As this description is analyzed as a quantifier construction, this might predict that conjunction reduction for sentences of the form (27) always is unproblematic. As explained below, I think it isn’t. Moreover, and also *pace* Breckenridge, I think that construing them as definite descriptions makes the wrong predictions regarding the modal profile of phenomenal ‘looks F’. Thus, I think that (i) is true, while Breckenridge predicts that it has a false reading:

(i) Red things might not have looked red.

Brogaard (2013; 2015) provides further reasons against construing *F* in ‘looks *F*’ as a manner adverbial.

15 In this wide sense, propositions are those things that essentially have, or determine, truth-conditions (including so-called “satisfaction” or “accuracy conditions”), and any mental state that has a proposition as its content is a propositional attitude.

16
*Inverse representationalism* holds that in experiences, phenomenal character determines representational content, and *strong representationalism* is the claim that, for perceptual experience, there is an equivalence relation between representational content and phenomenal character. There are also intentionalists who hold that only some, but not all, aspects of phenomenal character are to be explained by means of representational content (thus, for instance, Schellenberg (2008)).

17
^17^Travis (2004) implicitly denies the existence of a third use of ‘looks’, while Martin (2010) accepts that there is such a use, but construes it as ascribing certain properties to looks, properties such as splendidness. This appears to be motivated by parallel constructions involving sound or smell, but both the extent and the force of the parallel remain unclear to me – why exactly are we to conclude that, for instance, ‘looks red’ would have to be read as attributing redness to a look? Intentionalists often simply accept and sometimes utilize the existence of a third use of ‘looks’. Arguments and discussion are provided by Maund (1986) and more recently by Byrne (2009) and Brogaard (2013, 2014, 2015).

18 According to Byrne, experiences have singular contents, but why that would need to be displayed by means of a *de re* attitude report in this context, I do not know. Nor does it matter for present purposes.

19 Siegel (2010, 2011, 59ff,) accepts that there might not be ause of ‘looks’ in natural language that exclusively tracks what’s presented in visual experience, but argues that such publicly available looks wouldn’t be what’s relevant for the determination of experience content in any case. Moreover, she argues, there is no good reason to think that we couldn’t devise “a special, regimented sense of *looks* that did track what’s presented in visual phenomenology” (Siegel 2011, 63). For such aregimented sense, standard indexing would be true – by regimentation.

20 Yet another rival semantics for phenomenal ‘looks’ employing mind-independent relational properties can be derived from Brewer’s account of looks (cf. Brewer 2011, 118ff). Brewer construes looks in terms of an object’s visually relevant similarities with paradigms, relative to a point of view and other circumstances of perception.

21 And even if you don’t think experiences have contents, you could construe phenomenal looks reports as reporting on them: Gauker suggests interpreting such reports as telling us what assertions an experience would dispose an agent to make were she to be guided only by that experience (Gauker forthcoming, 28).

22 It should be noted, however, that this also requires further complicating the logical form of comparative looks-sentences. To have a chance at explaining the truth value data, these sentences need to be construed as *doubly context-sensitive*. As long as we think of (33) as saying that there is a salient, characteristic look that both Sam and bent things share, the sentence will be as false in all contexts as the sentence ‘Sam is bent’. Instead of

- (10 ^′^) ∃*y* (has(*o*, *y*) & (WL(*y*) & ID(C(*F*, WL, *c*), *y*))),

Martin thus (in effect) suggests the following logical form for comparative looks-sentences:

- (10 ^″^) ∃*y* (has(*o*, *y*) & (WL(*y*) & SIM(C(*F*, WL, *c*_1_), *y*, *c*_2_)))

where ‘SIM(*x*, *y*, *c*_2_)’ is the kind of similarity between *x* and *y* relevant in context *c*. (33) then is true if the look that Sam has is relevantly similar to whatever is the salient and characteristic look of the bent things in *c*. Martin explains: “The stick is similar to bent things simply with respect to how it strikes *me*, or the subjective bearing it has on me” (Martin 2010, 215). More precisely, looked at when half-way immersed in water, Sam is such that a speaker uttering (33) “enjoys a visual experience relative to which they would find the stick similar to a bent thing” (ibid., 221). It is here that the context dependence of SIM gets to do its work: the context somehow has shifted the similarity measure to something involving visual experiences of Sam. The need for double context dependence is due solely to the parsimonious account of looks. Even if we were to grant that all the relevant sentences are indeed comparative, no relational account needs to go doubly context dependent. Relational accounts of looks thus have the advantage of explaining the truth value data by means of a simpler semantics (cf. Glüer (2013) for a more detailed case in support of this claim). Nor is it the case that comparative sentences standardly are construed as doubly context dependent (or that there is any obvious reason for doing so). We therefore have good reason to reject Martin’s claim that “there is no reason from the semantics alone to attribute to objects more than the basic visible properties that we were committed to positing anyway” (Martin 2010, 222).

23 As an anonymous referee notes, ‘want’ might be taken to be another propositional attitude operator somewhat unhappy with that-clauses.

24 If you like, you can construe the example as one showing hyperintensionality, but that requires assuming a controversial semantics for proper names according to which co-referential proper names are co-intensional. I don’t subscribe to such a semantics (cf. Glüer and Pagin 2006), but nothing here will hang on the question of whether looks-contexts are intensional or hyperintensional.

25 Why not? The short answer is that what *guarantees* substitutivity salva veritate in attitude contexts is precisely either co-intensionality or co-hyperintensionality. Both entail co-extensionality. And the substitution behaviour of attitude contexts should be like that; if an operator is not sensitive to precisely differences in semantic content or meaning, it is not a propositional attitude operator, it seems to me. Phenomenal ‘looks’ is not: As we shall see, there are expressions that are not even co-extensional but substitutable in looks-contexts. For a bit more detail on this, see Glüer (2014), fn 38.

26 If you have difficulties reading ‘It looks as if *p*’ phenomenally, consider the following pair instead:

(i) Tom looks to be on the table in front of me.
(ii) Tim looks to be on the table in front of me.

27 If you think ‘rabbity’ and ‘ducky’ are not part of English, consider the following pair instead:

(i) Dubbit looks to be a rabbit.
(ii) Dubbit looks to be a duck.

In many expressions of the form ‘*x* looks to be *F*’ the infinitive can be deleted (cf. Brogaard 2015, 244). But this does not work for ‘looks to be a *G*’ where ‘*G*’ is a count noun.

28 The Gestalt an object takes is somehow determined by our visual system, of course. But whatever is responsible for Gestalt switching is not something we are aware of. What determines the intuitive truth values of looks-sentences, however, are changes we are eminently aware of.

29 Noë describes the acquiring the ability to see an object’s painter’s look as acquiring the ability of recovering an earlier stage of visual processing, i.e. recovering something like a two-dimensional image of the seen object (cf. for instance Noë (2004), 175ff), but I think that gets the phenomenology slightly wrong. An object’s painter’s look is as much a fully gestalted look as any other; having its painter’s look, the object looks like a very thin, flat material object (for more on this, see Glüer (2016a)).

30 An anonymous referee wonders whether phenomenal ‘looks’ might also be construed as itself ambiguous: Couldn’t it be the case that it sometimes works as a propositional attitude operator and at other times is used to ascribe relational properties to the objects of those attitudes? This hadn’t occurred to me, but I guess the question to ask in return is whether there really are any use data regarding phenomenal ‘looks’ forcing us to posit further ambiguity – data we cannot account for in terms of relational property ascription. So far, that does not seem to be the case to me. Note, too, that if the relational properties ascribed by phenomenal ‘looks’ are best construed as *phenomenal* relational properties, an ascription of such a property to an object *o* implies the existence of the experience *o* is the object of, and can thus be used to “report” on that experience (as noted above, Section 3).

31 Obviously, much more would need to be said about these properties. But this is not the place to do so. For a bit more on how I think phenomenal properties are best construed, see Glüer (2016b).

32 If this is right, one would also expect sentences containing ‘looks to be *F*’ to strike at least native speakers of English as entirely natural. While that might hold for native speakers from England, it does not quite generally seem to be the case, however.

33 Byrne himself calls the third use the “non-comparative use”.

34 What Byrne is interested in here is showing that phenomenal ‘looks’ does not index the content of experience. Here’s why: Mora is a baby. Like all naked mole rats, Mora was born looking old. But when you look at her, your experience might be perfectly veridical. Assuming that perfectly veridical experiences do not have false contents, Byrne concludes that the experience you can report by means of (58) does not ascribe being old to Mora. What it does ascribe is being bald, pink, and wrinkled.

35 A helpful analogy here would be personal pronouns. ‘Looks *F*’ expressions seem to pick up the property they ascribe from the context in as neat and systematic a way as personal pronouns pick up their referents.

36 For more on evaluation switchers, and their applications, see Glüer and Pagin (2006, 2012a, 2012b, 2014); Pagin and Westerståhl (2010c).

37 This objection has independently been raised by Elisabeth Coppock, Josep Macià, and Elia Zardini.
